# Supplementary material for: SPOP mutation induces replication over-firing by impairing Geminin ubiquitination and triggers replication catastrophe upon ATR inhibition
Source: Nat Commun. 2021 Oct 1;12:5779. doi: 10.1038/s41467-021-26049-6 (PMC8486843; doi:10.1038/s41467-021-26049-6)
Supplement: Supplementary file 1 — Supplementary Information [file 41467_2021_26049_MOESM1_ESM.pdf]

Supplementary Information

SPOP mutation induces replication over-firing by impairing Geminin ubiquitination and triggers replication catastrophe upon ATR inhibition

Supplementary Figures 1-6
Supplementary Tables 1-4

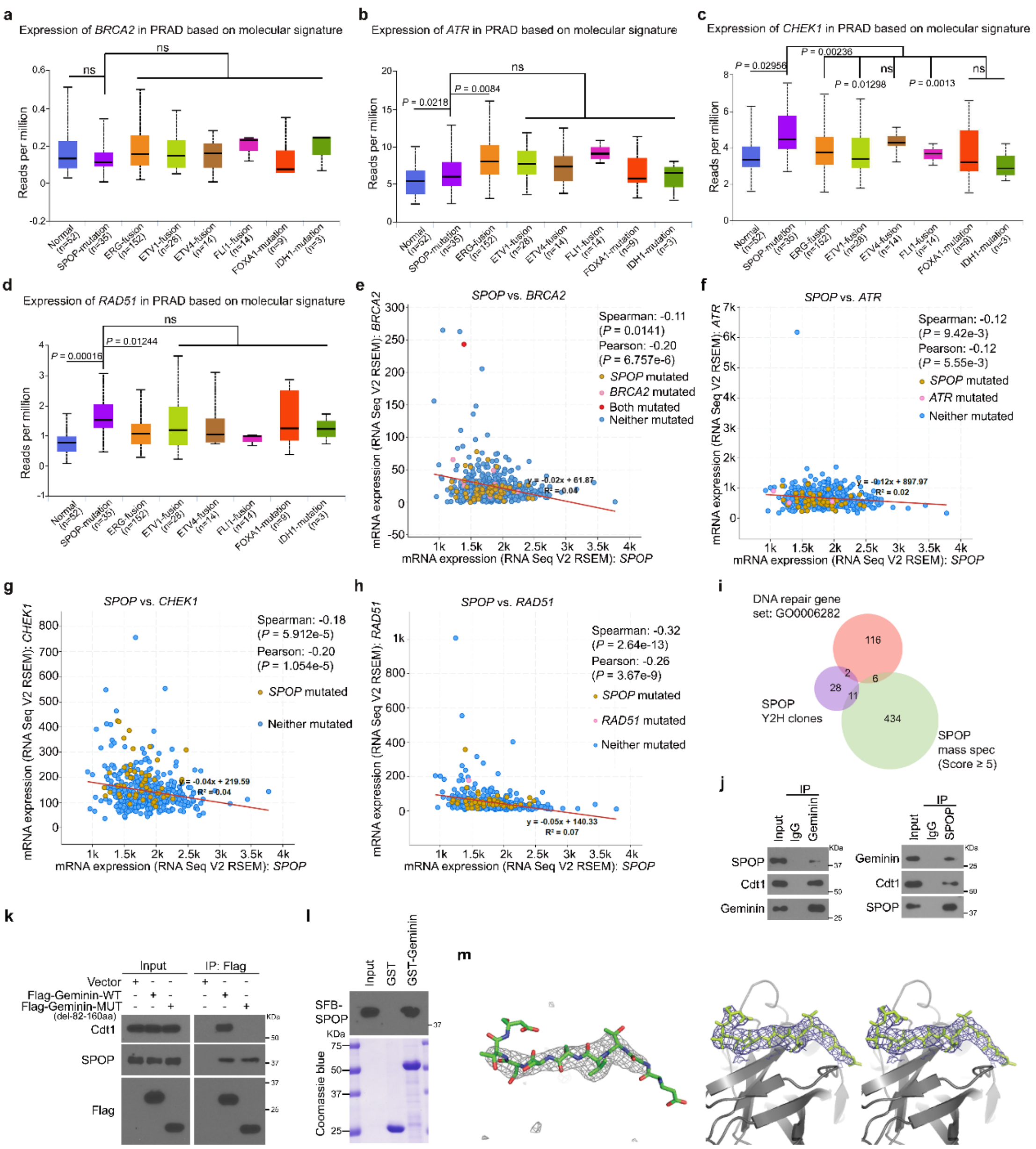

Supplementary Fig. 1 Geminin binds to SPOP and SPOP mutations have no effects on the mRNA levels of *BRCA2, ATR, CHEK1* and *RAD51* in prostate cancer patient specimens.

a-d The mRNA level of *BRCA2* (**a**), *ATR* (**b**), *CHEK1* (**c**) and *RAD51* (**d**) genes were compared between paired normal and tumor tissues from TCGA dataset. “n” represents the number of prostate cancer patients, boxplots shows minima, maxima and median. Two-tailed unpaired Student’s *t*-test; ns, not significant.

e-h The mRNA level of *BRCA2* (**e**), *ATR* (**f**), *CHEK1* (**g**) and *RAD51* (**h**) genes correlated to *SPOP* mRNA level. Two-tailed spearman's correlation and pearson correlation.

i Venn diagram showing the overlap of yeast two-hybrid screen data, mass spectrometry-based SPOP interactome, and the DNA repair gene set.

j Co-IP analysis of endogenous proteins in 293T cells using the indicated antibodies.

k 293T cells were transiently transfected with Geminin-WT or Geminin (82-160 aa deletion) and harvested for co-IP.
l GST pulldown assay using *in vitro* transcribed and translated SFB-SPOP protein and GST or GST-Geminin recombinant protein purified from *E. coli*.
m Left: 2*mFo*-*DFc* map of Geminin peptide in the X-ray structure of SPOP-MATH-Geminin peptide complex contoured at 1 σ level. Nine peptide residues were modeled in the electron density. Right: *Fo*-*Fc* omit map of the Geminin peptide in the X-ray structure of SPOP-MATH-Geminin peptide complex contoured at 3 σ level and shown in stereoview.
Source data are provided in this paper or Mendeley database (https://data.mendeley.com/datasets/8n7xt5rkhc/1). Similar results for **j, k, l** panels were obtained in two independent experiments.

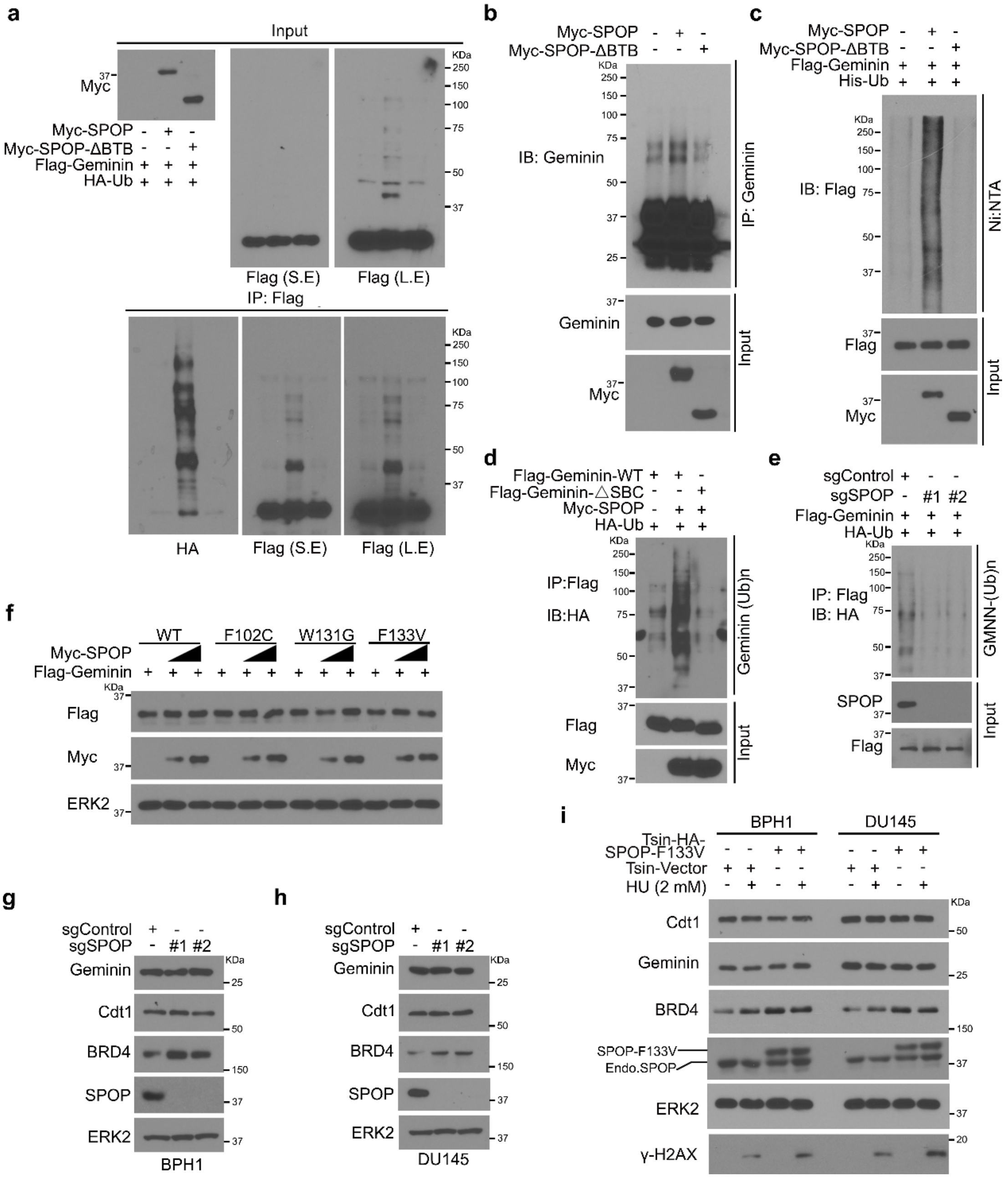

Supplementary Fig. 2 SPOP mediates Geminin non-degradative ubiquitination.

a 293T cells were transfected with the indicated plasmids and harvested for co-IP using anti-Flag-conjugated agarose beads. Ubiquitination from both input and IP samples were detected by indicated antibodies.

b 293T cells were transfected with the indicated plasmids and harvested for co-IP using anti-Geminin antibody and WB using indicated antibodies.

c 293T cells were transfected with the indicated plasmids and harvested for co-IP using Ni^{+2}–NTA beads.

d 293T cells were transfected with the indicated plasmids and harvested for co-IP using anti-Flag-conjugated agarose beads.

e 293T EV and SPOP-KO cells were transfected with the indicated plasmids and harvested for ubiquitination assay.
f 293T cells were transfected with the indicated plasmids and the level of indicated proteins was examined by WB.
g, h EV and SPOP-KO BPH1 cells (**g**) and DU145 cells (**h**) were harvested for WB with indicated antibodies.
i BPH1 and DU145 EV or SPOP F133V cells are treated with or without 2 mM HU for 4 h and harvested for WB with indicated antibodies.
Source data are provided in this paper or Mendeley database (https://data.mendeley.com/datasets/8n7xt5rkhc/1). Similar results for **a-i** panels were obtained in two independent experiments.

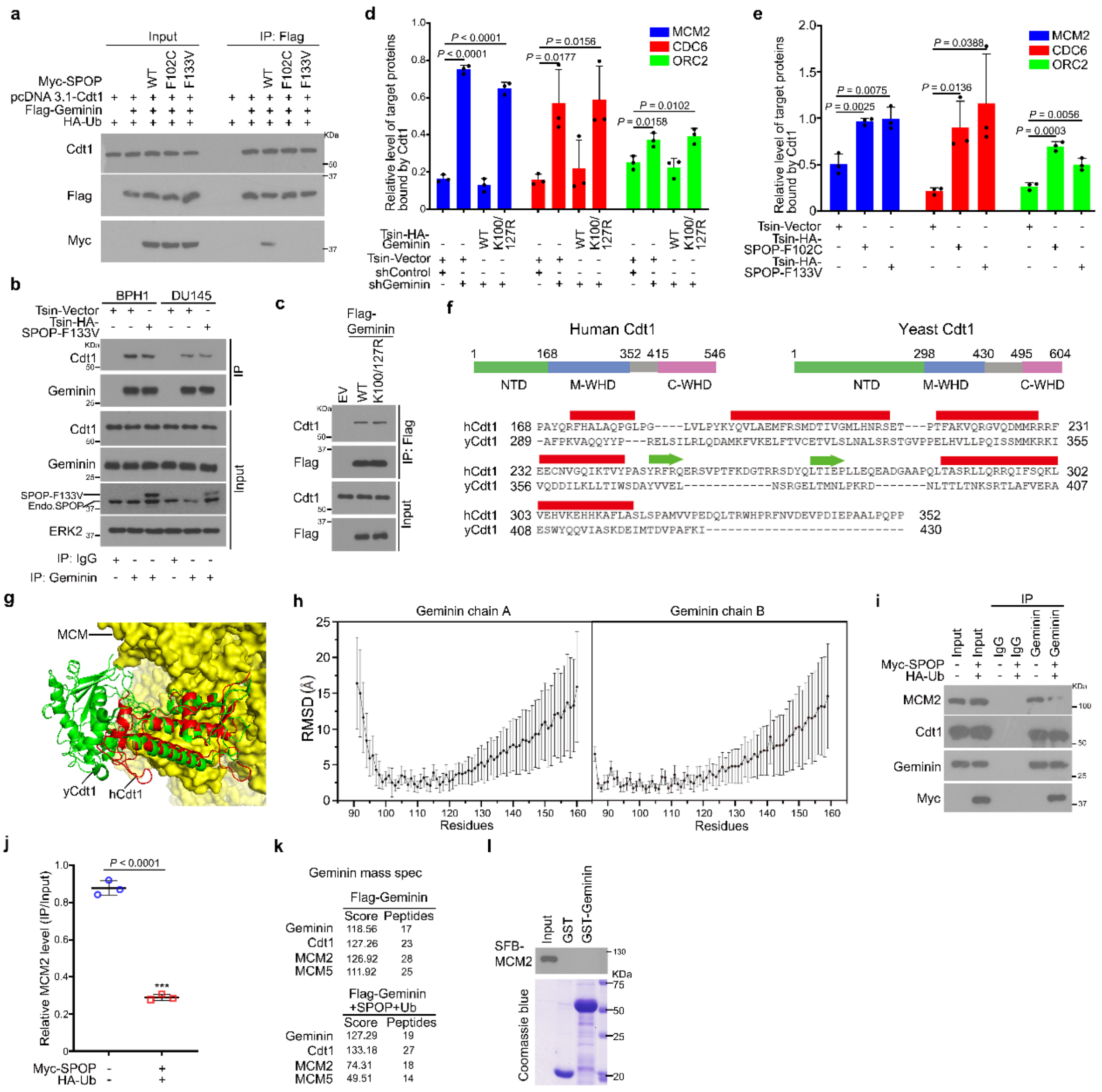

Supplementary Fig. 3 SPOP-mediated poly-ubiquitination of Geminin inhibits Cdt1 binding of MCM protein.

a 293T cells were transfected with the indicated plasmids and harvested for co-IP assay.

b DU145 and BPH1 cells stably expressing empty vector or HA-SPOP F133V mutant were harvested for co-IP analysis of the interaction between endogenous Geminin and Cdt1 proteins. Similar results were obtained in at least two independent experiments.

c 293T cells were transfected with Flag-tagged Geminin WT or K100/127R mutant and harvested for co-IP analysis.

d Quantification data from three repeats of co-IP assay as shown in Fig. 4a. Data are presented as the mean ± SD of three independent experiments (n=3). Two-tailed unpaired Student's *t*-test.

e Quantification data from three repeats of co-IP assay as shown in Fig. 4b. Data are presented as the mean ± SD of three independent experiments. Two-tailed unpaired Student's *t*-test.

f The middle winged-helix domains (M-WHD) in human and yeast Cdt1 (hCdt1 and yCdt1) show high structural similarities despite low sequence similarities. Red bar and green arrow indicate helical and beta-strand secondary structural elements in both Cdt1.
g The crystal structure of hCdt1 M-WHD (complexed to Geminin, PDB 2WVR) superimposed to the corresponding region of yCdt1 (bound to OCCM, PDB 6WGG) shows good fit and absence of any hCdt1-MCM steric clash.
h Molecular dynamics simulations using the crystal structure of hCdt1-Geminin (PDB 2WVR) show large fluctuations in the C-terminal residues of the two Geminin chains with respect to hCdt1. The graphs show the root mean square fluctuation (RMSF) of each Geminin residue position calculated with GROMACS in reference to the hCdt1-Geminin crystal structure after optimal fitting to hCdt1 backbone atoms. RMSF values were computed using 32,500 snapshots from the MD trajectory.
i, j 293T cells were transfected with the indicated plasmids and harvested for co-IP assay. Quantification data from three repeats were shown in (**j**). Data are presented as the mean ± SD of three independent experiments (n=3). Two-tailed unpaired Student's t-test.
k Mass spectrometry results from Geminin with or without transfection with Myc-SPOP and HA-Ub.
l GST pulldown assay using *in vitro* transcribed and translated SFB-MCM2 protein and GST or GST-Geminin recombinant protein purified from *E. coli*.
Source data are provided in this paper or Mendeley database (https://data.mendeley.com/datasets/8n7xt5rkhc/1). Similar results for **a, b, c, i** panels were obtained in two or three independent experiments.

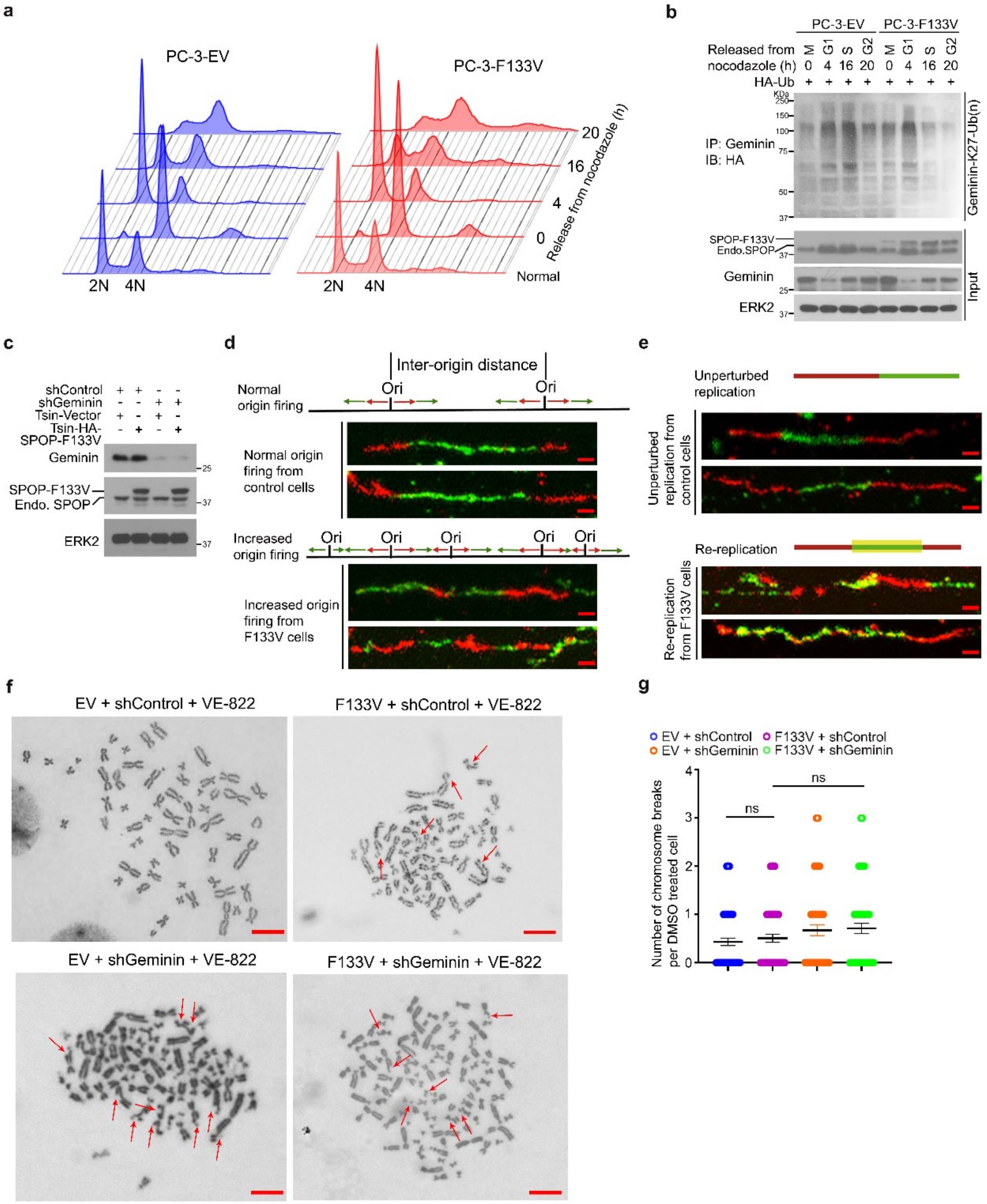

Supplementary Fig. 4 SPOP mutations increased replication catastrophe upon ATR inhibition.

a FACS-based cell cycle analysis of cells treated as indicated in Fig. 5c.

b EV- and F133V-expressing PC-3 cells transfected with HA-Ub were synchronized with nocodazole, released at different time points and harvested for IP and WB.

c WB analysis of indicated proteins in cells transfected as indicated in Fig. 5e-h.

d Diagram showing inter-origin distance and representative images of DNA replication detected by DNA fiber assay in control (top) and F133V-expressing PC-3 cells (bottom). Scale bar, 2 μm.

e Diagram showing re-replication and representative images of DNA replication detected by DNA fiber assay in control (top) and F133V-expressing PC-3 cells (bottom). Scale bar, 2 μm.
f Representative images of metaphase chromosomes with breaks used for the quantitative data shown in Fig. 5j. Red arrows point to the chromosome breaks. Scale bar, 10 μm.
g PC-3 cell lines were treated with DMSO for 24 h in parallel with experiments shown in Fig. 5j and harvested for karyotyping. Chromosome breaks per cell were quantified. More than 70 metaphases from 4 biological replicates were counted. Data are mean ± SEM. Two-tailed unpaired Student's *t*-test; ns, not significant.
Source data are provided in this paper or Mendeley database (https://data.mendeley.com/datasets/8n7xt5rkhc/1). Similar results for **b, c** panels were obtained in two independent experiments.

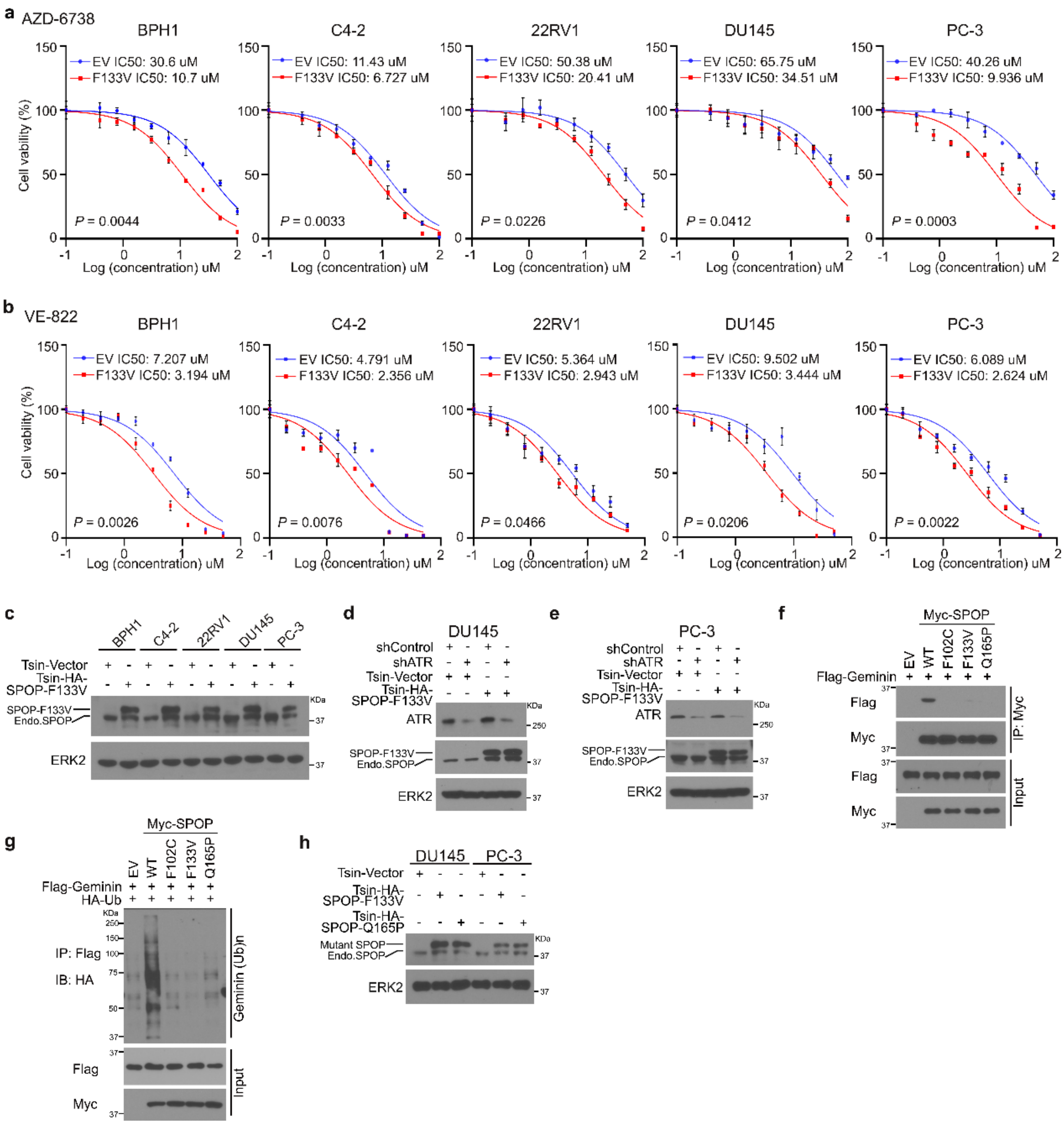

Supplementary Fig. 5 SPOP mutant cells are sensitive to ATR pathway inhibition.

a Dose surviving curve of AZD6738 in five prostatic cell lines expressing EV or SPOP F133V. Cells were treated with the indicated doses of AZD6738 for 24 h. Data are shown as the mean ± SD of three independent experiments (n=3 replicates/group). Two-tailed unpaired Student's *t*-test; ** $P < 0.01$, *** $P < 0.001$.

b Dose surviving curve of VE-822 in five prostatic cell lines expressing EV or SPOP F133V. Cells were treated with the indicated doses of VE-822 for 24 h. Data are shown as the mean ± SD of three independent experiments (n=3 replicates/group). Two-tailed unpaired Student's *t*-test; ** $P < 0.01$, *** $P < 0.001$.

c WB analysis of indicated proteins in cell lines expressing EV or SPOP F133V prior to the treatments as indicated in (a) and (b).

d, e WB analysis of indicated proteins in DU145 (d) and PC-3 cells (e) infected with indicated lentivirus as the cells used for Fig. 6b.

f 293T cells were transfected with indicated plasmids and harvested for co-IP assay.

g 293T cells were transfected with indicated plasmids and harvested for ubiquitination assay.
h WB analysis of indicated proteins in cells infected with indicated lentivirus as the cells used for Fig. 6d, e.
Source data are provided in this paper or Mendeley database (https://data.mendeley.com/datasets/8n7xt5rkhc/1). Similar results for **c-h** panels were obtained in two independent experiments.

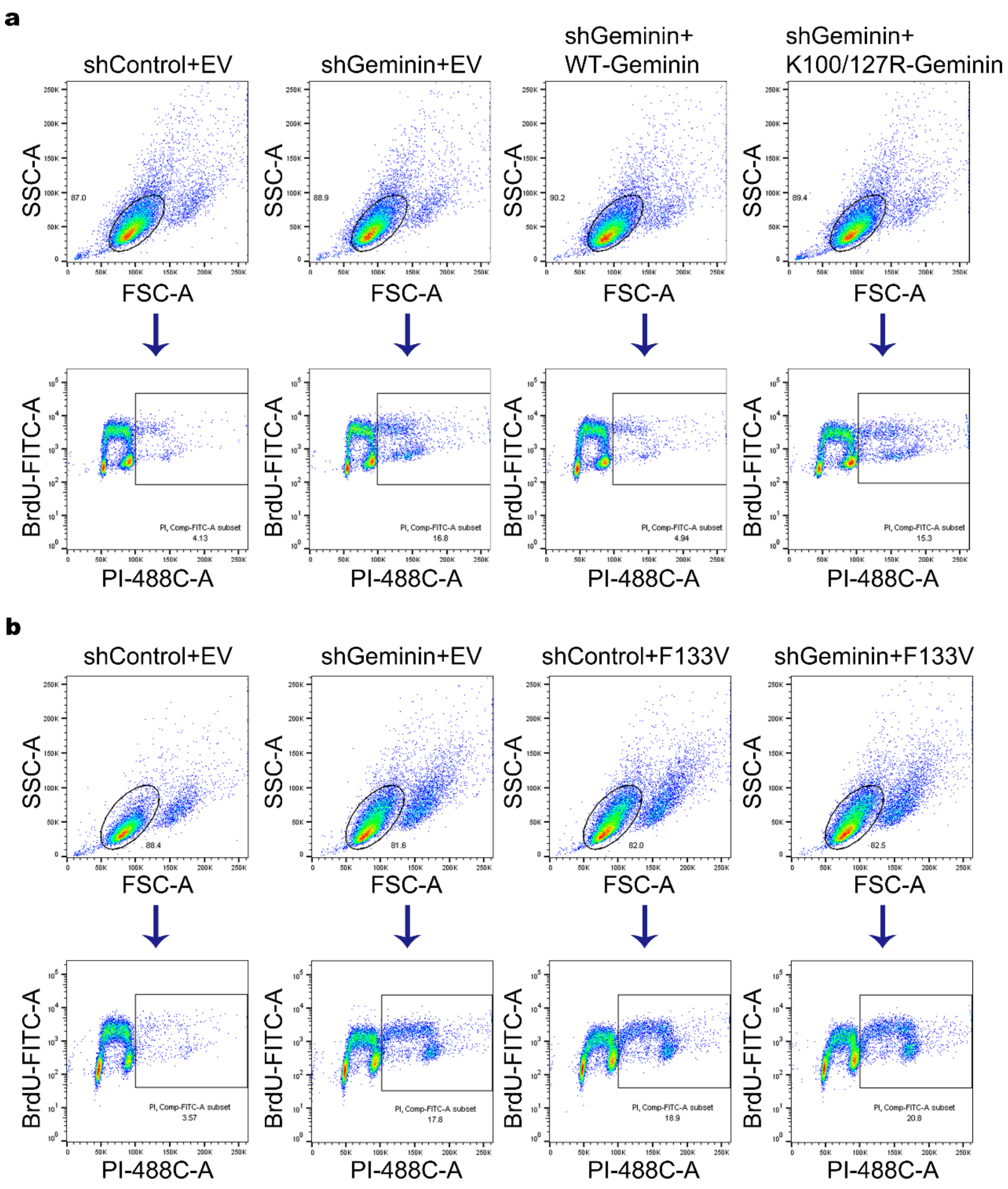

Supplementary Fig. 6 Related to Figure. 5 and Figure. 6. **Flow cytometry gating strategy.**

a BrdU incorporation measured by flow cytometry of PC-3 cells infected with lentivirus expressing control shRNA or Geminin-specific shRNA in combination with empty vector (EV), Geminin WT or K100/127R mutant related to Figure. 5e. Single cells gated by Forward Scatter (FSC-A) versus Side Scatter (SSC-A) plots were shown at top. The gated singlet population was displayed in a FITC-A (BrdU) versus 488C-A (DNA) plot. Cells with DNA content >4N were gated at bottom. 20,000 singlet events were collected for each experiment.

b BrdU incorporation measured by flow cytometry of PC-3 cells infected with lentivirus expressing EV or SPOP F133V in combination with control or Geminin-

specific shRNAs related to Figure. 6e. Single cells gated by Forward Scatter (FSC-A) versus Side Scatter (SSC-A) plots were shown at top. The gated singlet population was displayed in a FITC-A (BrdU) versus 488C-A (DNA) plot. Cells with DNA content >4N were gated at bottom. 20,000 singlet events were collected for each experiment.

Supplementary Table 1. The lists of DNA repair and replication genes

Regulation of DNA repair： GO:0006282			Regulation of DNA replication GO:0006275		
POLH	FUS	MRNIP	DBP	ENPP7	RAD17
PCNA	PPP4C	TEX15	STOML2	GLI1	INO80
WAS	CHEK1	PARP1	PCNA	CHEK2	WIZ
MAD2L2	XRCC1	BRCA1	BLM	BRCA2	TERF1
OTUB1	RTEL1	SPIRE2	NUCKS1	DNA2	TSPYL2
LOC107984078	CYREN	TWIST1	GLI2	SENP2	ZNF830
NUDT16	SIRT6	SPIRE1	ATG7	TICRR	EHMT2
RMI2	SETMAR	USP1	CCNA2	CDK1	JUN
FBH1	ZNF365	CEBPG	FBXO5	S100A11	RFC2
PARP3	TERF2	ERCC8	MSH3	CHTF8	RFC4
FIGN	NPAS2	WRAP53	DACH1	TP53	PAP
DDX11	RNF168	UBE2N	RFC3	TNFAIP1	UL42
POLQ	EYA3	EYA1	RFC5	CACYBP	NPM2
FMN2	SHLD1	ABRAXAS1	FBH1	STAG2	ESCO2
FANCB	NSD2	UBE2V2	ZMPSTE24	UCN	DBF4
SIRT1	APBB1	KLHL15	GMNN	RAC1	
RPA2	SLF2	KDM1A	ZBTB38	FGF10	
EYA4	H2AFX	FOXM1	LIG3	PDS5A	
HMGA2	EYA2	CSB-PGBD3	EGFR	MAS1	
EGFR	RTEL1-TNFRSF6B	DHX9	SMC1A	SMARCAL1	
RAD51AP1	FAM168A	NUDT16L1	GDF2	DONSON	
AXIN2	CBX8	AUNIP	CDT1	NBN	
TRIP12	RNF8	SHLD3	MSH6	HUS1	
RNF169	MGMT	PML	EREG	AICDA	
HELB	DTX3L	RAD51	ESCO1	GTPBP4	
TP53BP1	RAD52	TIGAR	ZRANB3	CDK2	
FIGNL2	PARP9	WDR70	ID3	CTC1	
HMGB1	PARG	OGG1	FBXW7	ATAD5	
SLF1	MAGEF1		ATR	TIPIN	
HDAC10	USP51		RBBP6	NUGGC	
RECQL5	FGF10		BCL6	USP37	
RPS3	RADX		CDC7	DBF4B	
BRCC3	UBE2V1		WAPL	CDC6	
HSF1	FIGNL1		ACVRL1	E2F7	
PRKCG	UIMC1		PPP2CA	HCRT	
CDK9	TFIP11		WRNIP1	TTF1	
UBR5	PRKDC		E2F8	CDAN1	
PARPBP	SHLD2		DSCC1	WDR18	

Supplementary Table 1 continued

Regulation of DNA repair：GO:0006282			Regulation of DNA replication GO:0006275		
TMEM161A	ERCC6		TIMELESS	ATRX	
BABAM1	CUL4A		MAP2K4	HRAS	
WRNIP1	SETD2		PPP2R1A	KCTD13	
SMCHD1	RIF1		MCIDAS	FGFR1	
KDM4D	TERF2IP		SLFN11	CCDC88A	
CGAS	MMS19		ATF1	MRE11	
TIMELESS	ACTR2		ANKRD17	STN1	
SPIDR	TRIM28		ZNF365	FAF1	
DEK	BABAM2		TERF2	DHX9	
UBQLN4	PPP4R2		CHTF18	SMC3	

Supplementary Table 2. SPOP putative binding proteins identified via Y2H screen

Gene name	Hits
BRD2	52
CHD3	35
CAPRIN1	31
ZMYND8	16
SETD2	12
BRD4	6
GLI3	6
DAXX	6
H2AFY	5
SRRM1	5
INF2	4
UBE2I	4
RANBP9	4
ZCCHC12	4
SPOP	4
NUDCD3	4
GCC2	4
PIAS3	4
RBFOX2	4
CBX4	4
AMOTL2	4
FAF1	3
GMNN	3
BRD3	2
GLI2	2
RBPJ	2

Supplementary Table 2 continued

Gene name	Hits
CHAF1A	2
DEK	2
NCOA3	1
PIAS1	1
TCOF1	1
SUMO1	1
RPRD2	1
MRE11A	1
LRCH4	1
KPNA5	1
HMGCS1	1
GMEB1	1
DHX15	1
CTDSPL2	1
CACUL1	1

Supplementary Table 3. SPOP putative binding partners identified via mass spectrometry

Gene name	Hits	Gene name	Hits	Gene name	Hits	Gene name	Hits
SPOP	106	CEP170	25	ATP5B	20	HIST1H1E	17
NUP153	69	NCL	25	CKAP5	20	HSPH1	17
TPR	67	ACACA	24	FASN	20	MATR3	17
NUP50	62	COPA	24	KPNA2	20	SERBP1	17
HNRNPK	41	HSPA8	24	LMNB2	20	HDLBP	16
DDX21	38	HSPD1	24	CUL3	19	ILF3	16
CAPRIN1	35	IGF2BP1	24	EIF4A1	19	KLHL7	16
KCTD12	35	TUBB2A	24	HSPA5	19	LMNA	16
KPNA6	34	PARP1	24	ILF2	19	NPM1	16
G3BP2	33	DLAT	23	KPNA5	19	RBM14	16
DHX9	32	HNRNPM	23	NONO	19	RPS18	16
KPNB1	32	DHX15	23	DDX1	18	RPS2	16
RPRD2	32	RTCB	22	EEF2	18	CCT2	15
G3BP1	30	XRCC6	22	IRS4	18	HSPA1L	15
TUBA1A	30	DDX5	21	MCM3	18	LARP1	15
DDX17	29	EEF1A1	21	PC	18	PHB2	15
PABPC1	29	ENO1	21	PKM	18	RPL4	15
HNRNPU	28	KPNA1	21	PRDX1	18	SF3B2	15
DBT	27	PTBP1	21	SF3B3	18	TCERG1	15
RPS3	27	RPS3A	21	VIM	18	TCP1	15
RPS4X	26	SUFU	21	ACTA2	17	TUFM	15
SYNCRIP	26	ATP5A1	20	EIF4A3	17	ZMYND8	15

Supplementary Table 3 continued

Gene name	Hits	Gene name	Hits	Gene name	Hits	Gene name	Hits
DHX30	14	HNRNPA0	11	RUVBL2	10	DDX6	8
HSPA1A	14	HNRNPA2B1	11	STAU1	10	DIS3	8
HSPA9	14	HNRNPF	11	ZC3H11A	10	DRG1	8
IQGAP1	14	HSP90AB1	11	ZC3HAV1	10	EIF2S2	8
MYBBP1A	14	IMPDH2	11	ZNF687	10	ELAVL1	8
RPL7	14	MCCC1	11	ANP32A	9	FBL	8
RPS14	14	PSMC3	11	ANXA2	9	GARS	8
XRCC5	14	RBM26	11	ATP5C1	9	GNL3	8
GLI3	14	RPS16	11	C14orf166	9	HCFC1	8
GTPBP4	14	RPS19	11	CCT4	9	HIST1H1A	8
SMC1A	14	RPS7	11	CYFIP1	9	HNRNPA1L2	8
DDX3Y	13	RSL1D1	11	GLYR1	9	HNRNPC	8
MAP4	13	RUVBL1	11	GNB2L1	9	HSPA2	8
MCCC2	13	SLC25A4	11	HNRNPA1	9	IGF2BP2	8
MTHFD1	13	SNRNP200	11	HNRNPH1	9	KPNA4	8
NUP107	13	TRIM24	11	HP1BP3	9	LUC7L2	8
PHB	13	UPF1	11	HSP90AB3P	9	NXF1	8
RBM45	13	XRN2	11	KIF22	9	PAICS	8
RPL3	13	YWHAE	11	KIF2A	9	PRPF19	8
RPL7A	13	INF2	11	MCM5	9	PSMD1	8
SF3B1	13	ATXN2L	10	NUP98	9	PSMD3	8
SPOPL	13	CALD1	10	PDCD11	9	PTRF	8
ACLY	12	DNAJA1	10	PHGDH	9	RPL21	8
AHCY	12	EEF1G	10	RPL10	9	RPL5	8
ATAD3A	12	EIF2A	10	RPL17	9	RPS17L	8
CKAP4	12	EPRS	10	RPL26L1	9	SF1	8
EIF4G1	12	FARSA	10	RPL8	9	SLC25A3	8
MOV10	12	HIST1H4A	10	RPL9	9	TUBB	8
PABPC4	12	HNRNPD	10	RPS15A	9	USP10	8
PCCA	12	HNRNPL	10	TPX2	9	XPO1	8
PCCB	12	HNRNPR	10	TUBB3	9	YBX1	8
PPP1R12A	12	IGF2BP3	10	U2AF2	9	PCNA	8
RPL23A	12	MCM7	10	SMC3	9	TRIM28	8
RPS9	12	NUP133	10	ABCF1	8	CCT3	7
SFPQ	12	PDHX	10	ACTB	8	CCT5	7
TUBB1	12	PSMC2	10	AIFM1	8	CCT7	7
UBA52	12	PSMD11	10	ATP1A1	8	COPB1	7
CACUL1	12	RBMX	10	ATP5O	8	CPSF7	7
FUS	12	RPL13	10	BUB3	8	DHX36	7
DCTN2	11	RPL6	10	DCTN1	8	DLD	7
HADHA	11	RPS6	10	DDX52	8	DNAJC7	7

Supplementary Table 3 continued

Gene name	Hits	Gene name	Hits	Gene name	Hits	Gene name	Hits
EFTUD2	7	DDX54	6	RPRD1B	6	DNAJA3	5
GLUD1	7	DKC1	6	RPS11	6	EIF2S3L	5
HIST1H1C	7	EIF2AK2	6	RPS24	6	EIF3A	5
HNRNPH2	7	ERP44	6	RPS25	6	EIF5A	5
HSP90AB2P	7	FAM98A	6	RTN4	6	EWSR1	5
KIFC1	7	FNBP4	6	SETD1A	6	EZR	5
LRRC59	7	GCC1	6	SHMT2	6	FAF2	5
NUP160	7	HAT1	6	SKIV2L2	6	FEN1	5
PDHB	7	HNRNPA3	6	SND1	6	FLNA	5
PRMT1	7	IRF2BP2	6	SRSF1	6	GSR	5
PRRC2A	7	KHDRBS1	6	TMPO	6	GTPBP1	5
PYCR2	7	KHSRP	6	TMPO	6	H1F0	5
RANBP2	7	KIF2C	6	TUBB4A	6	HADHB	5
RBM25	7	KIF5B	6	UBA1	6	HIST1H2AA	5
RECQL	7	LARP4B	6	VDAC2	6	HNRNPAB	5
RPL10A	7	LARS	6	YTHDC2	6	HNRNPCL3	5
RPL18	7	LMNB1	6	YTHDF2	6	HSD17B10	5
RPL31	7	LRRC47	6	YWHAB	6	HSD17B4	5
RPL38	7	NOP56	6	ZCCHC3	6	HSP90AA1	5
RPS13	7	NSUN2	6	ZRANB1	6	HSPA4	5
RPS8	7	NUDT21	6	CDK1	6	IARS	5
RXRB	7	NUFIP2	6	ABCE1	5	KCTD16	5
SARS2	7	PCBP1	6	ADAR	5	KPNA3	5
SEC24C	7	PGAM5	6	ALDH18A1	5	LRPPRC	5
SLC25A11	7	POLR2A	6	ALKBH5	5	MTHFD1L	5
SLC25A13	7	POLR2B	6	AP2A1	5	NAT10	5
SNRPA1	7	PPP1CA	6	ARMC8	5	NDUFA10	5
TUBB6	7	PRPF40A	6	CAPZA1	5	NKRF	5
USP7	7	PSMC5	6	CAT	5	NME1	5
VCP	7	PSMC6	6	CC2D1A	5	NMT1	5
DAXX	7	PSMD13	6	CCT8	5	NOP2	5
PPP2R1A	7	PUF60	6	CDK4	5	NTPCR	5
ANP32E	6	PURA	6	CKAP2	5	NUSAP1	5
ATXN2	6	RANGAP1	6	CLPB	5	OAT	5
CDC5L	6	RBBP4	6	CLTC	5	PFAS	5
CDKN2AIP	6	RCC2	6	CNP	5	PGM3	5
CEP170P1	6	RPL13A	6	COPG1	5	POLDIP3	5
COPB2	6	RPL15	6	CTNNA1	5	PRDX2	5
DARS	6	RPL23	6	DDX47	5	PRPF8	5
DDX3X	6	RPLP0P6	6	DHX57	5	PRPSAP1	5
DDX50	6	RPP30	6	DNAJA2	5	PSMA2	5

Supplementary Table 3 continued

Gene name	Hits	Gene name	Hits	Gene name	Hits	Gene name	Hits
PSMC1	5	SLC25A5	5	GMNN	5	ZC3H15	5
PSME3	5	SRSF3	5	RFC4	5	RPL22	5
PYCR1	5	SRSF9	5	RPL27	5	RPL19	5
RAN	5	STRAP	5	RPLP2	5	YWHAQ	5
RBM39	5	TK1	5	RPN1	5	SLC25A12	5
RCC1	5	TRIM25	5	RPRD1A	5	RPL14	5
RNMT	5	TSR1	5	RPS10P5	5	USP39	5
RPL11	5	TXN	5	RPS23	5	SERPINH1	5
RPL12	5	U2SURP	5	RRP1B	5		

**Supplementary Table 4 Resource information.** Hyperlinks need to be copy-pasted into the browser.		
**REAGENT or RESOURCE**	**SOURCE**	**IDENTIFIER**
**Antibodies**		
Mouse monoclonal anti-Geminin	Santa Cruz	Cat# sc-74456;<br>RRID: AB_1124963
Mouse monoclonal anti-Cdt1	Santa Cruz	Cat# sc-365305,<br>RRID: AB_10847805
Mouse monoclonal anti-MCM2	Santa Cruz	Cat# sc-373702,<br>RRID: AB_10917436
Mouse monoclonal anti-MCM3	Santa Cruz	Cat# SC-166940;<br>N/A
Mouse monoclonal anti-MCM4	Santa Cruz	Cat# sc-28317;<br>RRID: AB_627916
Mouse monoclonal anti-MCM7	Santa Cruz	Cat# sc-9966;<br>RRID: AB_627235
Mouse monoclonal anti-Cdc6	Santa Cruz	Cat# sc-9964;<br>RRID: AB_627236
Mouse monoclonal anti-ORC2	Santa Cruz	Cat# sc-32734;<br>RRID: AB_2157726
Mouse monoclonal anti-HA.11	Covance	Cat#MMS-101R;<br>RRID: AB_291262
Rabbit polyclonal anti-BRD4	Abcam	Cat# ab1228874;<br>N/A
Rabbit polyclonal anti-SPOP	Proteintech Group	Cat# 16750-1-AP;<br>N/A
Mouse monoclonal anti-Myc	Santa Cruz	Cat# sc-40;<br>RRID: AB_627268
Mouse monoclonal anti-Flag	Sigma	Cat # F-3165;<br>RRID: AB_259529
Mouse monoclonal anti-ERK2	Santa Cruz	Cat# sc-1647;<br>RRID: AB_627547
Rabbit monoclonal anti- Phospho Histone H2A.X (S139)	Cell Signaling Technology	Cat# 9718;<br>RRID: AB_2118009
Goat anti-Rabbit IgG (H+L) Alexa Fluor 594	Thermo Fisher	Cat # A11037;<br>RRID: AB_2534095
Peroxidase IgG Fraction Monoclonal Mouse Anti-Rabbit IgG	Jackson ImmunoResearch	Cat# 211-032-171;<br>RRID: AB_2339149
Peroxidase AffiniPure Goat Anti-Mouse IgG	Jackson ImmunoResearch	Cat#115-035-174;<br>RRID: AB_2338512
Rat monoclonal anti-BrdU	Abcam	Cat# ab6326;<br>RRID: AB_305426
Mouse monoclonal anti-BrdU	BD Bioscience	Cat# 347580;<br>RRID: AB_400326

**Supplementary Table 4 continued**		
Goat anti-Mouse IgG (H+L) Alexa Fluor 488	Thermo Fisher	Cat # A11029;<br>RRID: AB_138404
Alexa Fluor 488 Goat Anti-Rat IgG (H+L)	Life Technologies	Cat# A-11006,<br>RRID: AB_2534074
Mouse-IgGκ BP-FITC	Santa Cruz	Cat# sc-516140,<br>RRID: N/A
**Bacterial and Virus Strains**		
E. coli DH5α	Thermo Fisher	Cat#18258012
E. coli: BL21(DE3)	New England Biolabs	Cat#C2527H
lentivirus-expressing shNS control (human)	Sigma-Aldrich	SHCLNG-NM_001
lentivirus-expressing shGeminin (human)	Sigma-Aldrich	SHCLNV-NM_015895
lentivirus-expressing shATR (human)	Sigma-Aldrich	SHCLND-NM_001184
**Chemicals, Peptides and Recombinant Proteins**		
MG132	EMD Chemicals	Cat# 474790
Lipofectamine 2000 reagent	Thermo Fisher	Cat# 11668500
Protein A/G agarose beads	Thermo Fisher	Cat# 20423
5-lodo-2'-deoxyuridine Idoxuridine (IDU)	Cayman	Cat# 20222-1
5-Chloro-2'-deoxyuridine (CIDU)	Cayman	Cat# 18155-50
VE-822	TargetMol	Cat# 1232416-25-9
AZD6738	Selleck Chemicals	Cat# S7693
Matrigel matrix	BD Bioscience	Cat# 354234
Geminin SBC peptides	Mujin Biotech	Cat# MJ190704
LB broth, Miller (Luria-Bertani)	Difco	Cat#244610
LB agar, Miller (Luria Bertani)	Difco	Cat#244520
M9 minimal salts 5x	Difco	Cat#248510
Ampicillin	Gold Biotechnology	Cat#A-301-100
IPTG	RPI	Cat#32115
Tris	Bio-Rad	Cat#161-0716
Sodium phosphate monobasic	Fisher	Cat#BP329-1
Sodium phosphate dibasic	Fisher	Cat#S373-3
Potassium phosphate monobasic	Fisher	Cat#BP362-1
Sodium chloride	Fisher	Cat#S271-10
Potassium chloride	Alfa Aesar	Cat#A11662
Imidazole	Acros	Cat#122025000
Nickel sulfate	Alfa Aesar	Cat#12514
EDTA	Acros	Cat#40993-0010
PMSF	MP	Cat#195381
Dithiothreitol	BioWorld	Cat#705325

Supplementary Table 4 continued		
^{15}N, ammonium chloride	Sigma	Cat#299251-1G
Deuterium oxide	CIL	Cat#DLM-4-1000
Isogro ^{15}N powder growth medium	Sigma	Cat#606871
PEG 3350	Hampton	Cat#HR2-527
Xylitol	Sigma	Cat#113K0033
**Critical Commercial Assays**		
KOD Plus Mutagenesis Kit	Toyobo	Cat# F0936K
NE-PER™ Nuclear and Cytoplasmic Extraction Reagents	Thermo Fisher	Cat# 78833
Red Blood Cell (RBC) Lysis Buffer	BioLegend	Cat# 422401
SignalStain® DAB Substrate Kit	Cell Signaling Technology	Cat# 8059
QIAquick Gel Extraction Kit	Qiagen	Cat # 28704

**Primer List**	
His-SPOP-MATH-F	GGAATTCCATATGGTAGTGAAATTCTCCTACATGT
His-SPOP-MATH-R	CGCGGATCCATTACCCAGAGGTCTCCAAGAC
Geminin 27K-R-F:	CGGATGATTCAGCCTTCTGCA
Geminin 27K-R-R:	CAGAGTTCTTCTTGGGACAGA
Geminin 50K-R-F:	CGGAGGAAACATCGGAATGAC
Geminin 50K-R-R:	GGACAAGCCTGCGGACAGCTC
Geminin 100K-R-F:	CGGGAAGTGGCAGAAAAACGG
Geminin 100K-R-R:	CCAATACTGAGAGGATGGATT
Geminin 127K-R-F:	CGGGACAATGAAATTGCCCGC
Geminin 127K-R-R:	TTGTTCAATTTCTTTATGAAG
Geminin-DEL-SBC-F	GATGCAAAGCCATGTATATGA
Geminin-DEL-SBC-R	AGTTCCTTCAGCACACGTGCC
SPOP sgRNA-F1	CACCGCAAGCTTACCCTCTTCTGCG
SPOP sgRNA-R1	AAACCGCAGAAGAGGGTAAGCTTGC
SPOP sgRNA-F3	CACCGGTCATCAGGGAGAAGCCCGT
SPOP sgRNA-R3	AAACACGGGCTTCTCCCTGATGACC
pTsin-Myc-SPOP-F	CGC GGATCC ATG GCA TCA ATG CAG AAG CTG A
pTsin-Myc-SPOP-R	CCG CTCGAGTTA GGA TTG CTT CAG GCG TTT GCG T
Tsin-GMNN-ASCI-f	AGGCGCGCCT ATGAATCCCAGTATGAAGCA
Tsin-GMNN--XHOI-r	CCG CTCGAG TTA TATACATGGCTTTGCATCCG
Geminin 202S-F-F:	TTTACGGATGCAAAGCCATG
Geminin 202S-F-R:	GGAAGATACAGTTCCTTCAG

**Deposited Data**		
The whole-genome sequencing data	(Ren et al., 2017)[1]	EGAS00001000888

Supplementary Table 4 continued

The Cancer Genome Atlas (TCGA) prostate cancer dataset	(Cancer Genome Atlas Research, 2015)[2]	Cancer Genome Atlas Research Network
Gene Set: regulation of DNA replication	Gene Ontology	GO:0006275
Gene Set: regulation of DNA repair	Gene Ontology	GO:0006282
SPOP yeast two-hybrid screen data	This paper	N/A
Mass spectrometry-based SPOP interactome	This paper	N/A
Mass spectrometry-based Geminin ubiquitination	This paper	N/A
Raw data and images	This paper and Mendeley Data	https://data.mendeley.com/datasets/8n7xt5rkhc/1
2WVR	(De Marco et al., 2009)[3]	Protein Data Bank
6WGG	(Yuan et al., 2020)[4]	Protein Data Bank
7KLZ	This paper	Protein Data Bank
**Experimental Models: Cell Lines**		
Human: C4-2	Uro Corporation	N/A
Human: PC-3	ATCC	CRL-1435
Human: DU145	ATCC	HTB-81
Human: HEK293T	ATCC	CRL-11268
Human:22Rv1	ATCC	CRL-2505
Human: BPH-1	Dr. S. W. Hayward	(Hayward et al., 1995)[5]
**Oligonucleotides**		
Refer to Primer List above for primer sequences	IDT	N/A
**Recombinant DNA**		
psPAX2	Addgene	Cat# 12260
pMD2.G	Addgene	Cat# 12259
pCMV-HA	Addgene	Cat# 32530
Puro. Cre-EV (Tsin-EV)	Addgene	Cat# 17408
pEnter-Flag-Geminin	Vigene Biosciences	Cat# CH868616
pcDNA3.1-Cdt1	Addgene	Cat# 72681
Tsin-HA-SPOP-WT, F102C, F133V, Q165P	This paper	N/A
pCMV- Myc-SPOP-Y87C, Y87N, F102C, S119N, F125V, K129N, W131C, W131G, F133L, F133V, K134N	This paper	N/A
pCMV-Myc-SPOP-ΔBTB	This paper	N/A
pCMV-Myc-SPOP-ΔMATH	This paper	N/A

Supplementary Table 4 continued

**Software and Algorithms**		
ImageJ	NIH	RRID: SCR_001935 https://imagej.nih.gov/ij/
FlowJo	FlowJo LLC	RRID: SCR_008520 https://www.flowjo.com/solutions/flowjo
GraphPad Prism 8.0	GraphPad, Inc.	RRID: SCR_002798 http://www.graphpad.com/
ZEN Digital Imaging for Light Microscopy	Carl Zeiss	RRID: SCR_013672 http://www.zeiss.com/microscopy/en_us/products/microscope-software/zen.html#introduction
PHENIX	University College London	https://phenix-online.org/
PyMOL	The PyMOL Molecular Graphics System	https://pymol.org/2/
NMRView	One Moon Scientific, Inc.	https://www.onemoonscientific.com/
NMRPipe	Institute for Bioscience and Biotechnology Research	https://www.ibbr.umd.edu/nmrpipe/index.html
HKL2000	HKL2000 Research Inc.	https://www.hkl-xray.com/
GROMACS	University of Groningen	https://www.gromacs.org/

Supplementary References

1. Ren S*, et al.* Whole-genome and transcriptome sequencing of prostate cancer identify new genetic alterations driving disease progression. *Eur Urol* **73**, 322-339 (2017).

2. Cancer Genome Atlas Research N. The Molecular Taxonomy of Primary Prostate Cancer. *Cell* **163**, 1011-1025 (2015).

3. De Marco V*, et al.* Quaternary structure of the human Cdt1-Geminin complex regulates DNA replication licensing. *Proc Natl Acad Sci U S A* **106**, 19807-19812 (2009).

4. Yuan Z*, et al.* Structural basis of Mcm2-7 replicative helicase loading by ORC-Cdc6 and Cdt1. *Nat Struct Mol Biol* **24**, 316-324 (2017).

5. Hayward SW, Dahiya R, Cunha GR, Bartek J, Deshpande N, Narayan P. Establishment and characterization of an immortalized but non-transformed human prostate epithelial cell line: BPH-1. *In Vitro Cell Dev Biol Anim* **31**, 14-24 (1995).
