# Supplementary material for: SPOP mutation induces replication over-firing by impairing Geminin ubiquitination and triggers replication catastrophe upon ATR inhibition
Source: Nat Commun. 2021 Oct 1;12:5779. doi: 10.1038/s41467-021-26049-6 (PMC8486843; doi:10.1038/s41467-021-26049-6)
Supplement: Supplementary file 3 — Reporting Summary [file 41467_2021_26049_MOESM3_ESM.pdf]

nature portfolio

Corresponding author(s): Haojie Huang, NCOMMS-21-03998

Last updated by author(s): Jul 29, 2021

Reporting Summary

Nature Portfolio wishes to improve the reproducibility of the work that we publish. This form provides structure for consistency and transparency in reporting. For further information on Nature Portfolio policies, see our Editorial Policies and the Editorial Policy Checklist.

Statistics

For all statistical analyses, confirm that the following items are present in the figure legend, table legend, main text, or Methods section.

n/a	Confirmed	
☐	☒	The exact sample size (*n*) for each experimental group/condition, given as a discrete number and unit of measurement
☐	☒	A statement on whether measurements were taken from distinct samples or whether the same sample was measured repeatedly
☐	☒	The statistical test(s) used AND whether they are one- or two-sided<br>*Only common tests should be described solely by name; describe more complex techniques in the Methods section.*
☐	☒	A description of all covariates tested
☐	☒	A description of any assumptions or corrections, such as tests of normality and adjustment for multiple comparisons
☐	☒	A full description of the statistical parameters including central tendency (e.g. means) or other basic estimates (e.g. regression coefficient) AND variation (e.g. standard deviation) or associated estimates of uncertainty (e.g. confidence intervals)
☐	☒	For null hypothesis testing, the test statistic (e.g. *F*, *t*, *r*) with confidence intervals, effect sizes, degrees of freedom and *P* value noted<br>*Give P values as exact values whenever suitable.*
☐	☒	For Bayesian analysis, information on the choice of priors and Markov chain Monte Carlo settings
☐	☒	For hierarchical and complex designs, identification of the appropriate level for tests and full reporting of outcomes
☒	☐	Estimates of effect sizes (e.g. Cohen's *d*, Pearson's *r*), indicating how they were calculated

Our web collection on statistics for biologists contains articles on many of the points above.

Software and code

Policy information about availability of computer code

Data collection	DNA fiber: Carl Zeiss ZEN2; Cryo-EM: SerialEM; NMR Spectroscopy: Bruker AVANCE III;
Data analysis	Graphing and statistical analysis: GraphPad Prism 8.0; Cryo-EM: MotionCor2, CryoSPARC v2.15; Model Building: COOT v0.8.9.2, PHENIX v1.18.2-3874; Structure Visualization: PyMol v2.3.2, wwPDB validation server; Molecular Dynamics Simulations: GROMACS; NMR spectroscopy: NMRPipe,NMRViewJ (OneMoon Scientific, Inc.); Gel Quantification: ImageJ v1.53e; Flow Cytometry: FlowJo 10.4 LLC; LC-MS analysis: EASY-nLC 1000 system (Thermo Fisher Scientific, Odense, Denmark), Orbitrap Elite mass spectrometer (Thermo Fisher Scientific, Bremen, Germany), Protein Discoverer (Version 2.4.1.15, Thermo Fisher Scientific) and Mascot (Version 2.7.0, Matrix Science)

For manuscripts utilizing custom algorithms or software that are central to the research but not yet described in published literature, software must be made available to editors and reviewers. We strongly encourage code deposition in a community repository (e.g. GitHub). See the Nature Portfolio guidelines for submitting code & software for further information.

Data

Policy information about availability of data

All manuscripts must include a data availability statement. This statement should provide the following information, where applicable:

- Accession codes, unique identifiers, or web links for publicly available datasets
- A description of any restrictions on data availability
- For clinical datasets or third party data, please ensure that the statement adheres to our policy

The raw data for western blot generated in this study have been deposited in the Mendeley database. The atomic coordinates for the protein structure presented in this publication are deposited in the Protein Data Bank under accession code 7KLZ. Already published protein structures used in this study can be found in the Protein Data Bank under accession codes: 2WVR and 6WGG. The mass spectrometry proteomics data have been deposited to the ProteomeXchange Consortium

(http://proteomecentral.proteomexchange.org) via the iProX partner repository with the dataset identifier PXD027915 and PXD028210.

Field-specific reporting

Please select the one below that is the best fit for your research. If you are not sure, read the appropriate sections before making your selection.

- [x] Life sciences
- [ ] Behavioural & social sciences
- [ ] Ecological, evolutionary & environmental sciences

For a reference copy of the document with all sections, see nature.com/documents/nr-reporting-summary-flat.pdf

Life sciences study design

All studies must disclose on these points even when the disclosure is negative.

Sample size	An N of 5 to 10 mice was used for each condition in each experiment, based on standard practice in the field and prior experience working with the specified mouse models. For other experiments, no sample size calculation was performed. We chose the sample size to obtain a representative number of cells from several independent experiments to get enough statistical power.
Data exclusions	No data were excluded from the analyses.
Replication	Data are presented as the mean ± SD. Replicates of experiments are specified in figure legends
Randomization	In mouse experiments, all samples and animals are randomly divided into different experimental groups as indicated. For other studies, experiments were performed in large cell line populations, and randomization was therefore not appropriate.
Blinding	For mouse study, investigators were blinded to group assignment during data collection and analysis. For flow cytometry and DNA fiber studies, the researchers were blinded during data collection. For western blot and protein stuctural studies, researchers were not blinded because it was not practically feasible to conduct truly blinded experiments with the available laboratory staff.

Reporting for specific materials, systems and methods

We require information from authors about some types of materials, experimental systems and methods used in many studies. Here, indicate whether each material, system or method listed is relevant to your study. If you are not sure if a list item applies to your research, read the appropriate section before selecting a response.

Materials & experimental systems

n/a	Involved in the study	
[ ]	[x]	Antibodies
[ ]	[x]	Eukaryotic cell lines
[x]	[ ]	Palaeontology and archaeology
[ ]	[x]	Animals and other organisms
[x]	[ ]	Human research participants
[x]	[ ]	Clinical data
[x]	[ ]	Dual use research of concern

Methods

n/a	Involved in the study	
[x]	[ ]	ChIP-seq
[ ]	[x]	Flow cytometry
[x]	[ ]	MRI-based neuroimaging

Antibodies

Antibodies used

1. anti-Geminin (Santa Cruz, Cat# sc-74456, RRID: AB_1124963),
2. anti-Cdt1 (Santa Cruz, Cat# sc-365305, RRID: AB_10847805),
3. anti-MCM2 (Santa Cruz, Cat# sc-373702, RRID: AB_10917436),
4. anti-MCM3 (Santa Cruz, Cat# SC-166940),
5. anti-MCM4 (Santa Cruz, Cat# sc-28317, RRID: AB_627916),
6. anti-MCM7 (Santa Cruz, Cat# sc-9966, RRID: AB_627235),
7. anti-Cdc6 (Santa Cruz, Cat# sc-9964, RRID: AB_627236),
8. anti-ORC2 (Santa Cruz, Cat# sc-32734, RRID: AB_2157726),
9. anti-HA.11 (Covance, Cat#MMS-101R; RRID: AB_291262),
10.anti-BRD4 (Abcam, Cat# ab1228874),
11. anti-SPOP (Proteintech Group, Cat# 16750-1-AP),
12. anti-Myc (Santa Cruz, Cat# sc-40, RRID: AB_627268),
13. anti-Flag (Sigma, Cat # F-3165, RRID: AB_259529),
14. anti-ERK2 (Santa Cruz, Cat# sc-1647, RRID: AB_627547),
15. anti- Phospho Histone H2A.X (S139) (Cell Signaling, Cat# 9718, RRID: AB_2118009),
16. anti-Rabbit IgG (H+L) Alexa Fluor 594 (Thermo Fisher, Cat # A11037, RRID: AB_2534095),
17. anti-Rabbit IgG (Jackson ImmunoResearch, Cat# 211-032-171, RRID: AB_2339149),

18. anti-Mouse IgG (Jackson ImmunoResearch, Cat#115-035-174, RRID: AB_2338512),
19. anti-BrdU (Abcam, Cat# ab6326, RRID: AB_305426),
20. anti-BrdU (BD Bioscience, Cat# 347580, RRID: AB_400326),
21. anti-Mouse IgG (H+L) Alexa Fluor 488 (Thermo Fisher, Cat # A11029, RRID: AB_138404),
22. anti-Rat IgG (H+L) Alexa Fluor 488 (Life Technologies, Cat# A-11006, RRID: AB_2534074),
23. Mouse-IgGκ BP-FITC (Santa Cruz, Cat# sc-516140)

Validation	All of the antibodies used in this study were validated for use in human specimens by the manufacturers and for the respective methods used in this manuscript (please see home pages of respective manufacturers using catalogue numbers or RRIDs of the antibodies provided above).

Eukaryotic cell lines

Policy information about cell lines

Cell line source(s)	The immortalized human embryonic kidney cell line 293T and prostate cancer cell lines PC-3, DU145 and 22RV1 were purchased from ATCC (Manassas, VA). C4-2 cells were purchased from Uro Corporation (Oklahoma City, OK). BPH1 cells were kindly provided by Dr. Simon Hayward.
Authentication	The cell lines were authenticated periodically via STR profiling (IDEXX BioResearch).
Mycoplasma contamination	All cell lines were tested negative of mycoplasma contamination.
Commonly misidentified lines (See ICLAC register)	No

Animals and other organisms

Policy information about studies involving animals; ARRIVE guidelines recommended for reporting animal research

Laboratory animals	6-8 weeks old SCID male mice were used for xenograft study as described in the Methods section. All mice were housed under standard conditions at room temperature with a 12 h light/dark cycle with access to food and water ad libitum and maintained under pathogen-free conditions. The animal study was approved by the Institutional Animal Care and Use Committee (IACUC) at the Mayo Clinic.
Wild animals	No
Field-collected samples	No
Ethics oversight	The animal study was approved by the Institutional Animal Care and Use Committee (IACUC) at the Mayo Clinic

Note that full information on the approval of the study protocol must also be provided in the manuscript.

Flow Cytometry

Plots

Confirm that:

- [x] The axis labels state the marker and fluorochrome used (e.g. CD4-FITC).
- [x] The axis scales are clearly visible. Include numbers along axes only for bottom left plot of group (a 'group' is an analysis of identical markers).
- [x] All plots are contour plots with outliers or pseudocolor plots.
- [x] A numerical value for number of cells or percentage (with statistics) is provided.

Methodology

Sample preparation	PC-3 and 293T cells were treated with 2 mM thymidine for 24 h and released into regular culture medium for 3 h. After washing with PBS three times, cells were released into regular medium for another 9 h, after which cells were blocked by L-Mimosine (300 µM final concentration) for 24 h and released into regular medium. At the indicated time points after final release, cells were harvested for cell cycle profiling. For BrdU incorporation, PC-3 cells were treated with 10 µM BrdU for 30–45 min before harvest.
Instrument	BD CANTOII were used for cell cycle analysis.
Software	FlowJo
Cell population abundance	Above 20,000 cells were counted per sample.
Gating strategy	Single cells gated by Forward Scatter (FSC-A) versus Side Scatter (SSC-A) plots were shown at the top. The gated singlet

population was displayed in a FITC-A (BrdU) versus 488C-A (DNA) plot. Cells with DNA content >4N were gated at bottom. 20,000 singlet events were collected for each experiment.

☒ Tick this box to confirm that a figure exemplifying the gating strategy is provided in the Supplementary Information.
